# Energetics and dynamics of the proton shuttle of carbonic anhydrase II

**DOI:** 10.1007/s00018-023-04936-z

**Published:** 2023-09-09

**Authors:** Heiner N. Raum, Suzanne Zoë Fisher, Ulrich Weininger

**Affiliations:** 1 Institute of Physics, Biophysics, Martin-Luther-University Halle-Wittenberg, 06120 Halle (Saale), Germany; 2 Department of Biology and Lund Protein Production Platform, Lund University, Sölvegatan 35, 22362 Lund, Sweden; 3 Scientific Activities Division, European Spallation Source ERIC, P.O. Box 176, 22100 Lund, Sweden

**Keywords:** Enzyme catalysis, NMR spectroscopy, Protein dynamics, Proton shuttling, Proton transport

## Abstract

Human carbonic anhydrase II catalyzes the reversible reaction of carbon dioxide and water to form bicarbonate and a proton. His64-mediated proton shuttling between the active site and the bulk solvent is rate limiting. Here we investigate the protonation behavior of His64 as well as its structural and dynamic features in a pH dependent way. We derive two p*K*_a_ values for His64, 6.25 and 7.60, that we were able to assign to its inward and outward conformation. Furthermore, we show that His64 exists in both conformations equally, independent of pH. Both conformations display an equal distribution of their two neutral tautomeric states. The life time of each conformation is short and both states display high flexibility within their orientation. Therefore, His64 is never static, but rather poised to change conformation. These findings support an energetic, dynamic and solution ensemble-based framework for the high enzymatic activity of human carbonic anhydrase II.

**Supplementary Information:**

The online version contains supplementary material available at 10.1007/s00018-023-04936-z.

## Introduction

Human carbonic anhydrase II (HCAII) is a highly efficient enzyme that catalyzes the reversible conversion of carbon dioxide and water to form bicarbonate and a proton [1, 2]. The catalytic step is performed by a zinc-bound hydroxide or water, while the rate limiting step is the proton transfer between the active site and the buffer or bulk solvent, which is mediated by a hydrogen-bonded network of water molecules and the proton shuttle residue His64 [3–5]. Substituting His64 by Ala reduces the overall turnover of HCAII by factor 20 [4].

The reaction mechanism follows a ping-pong (2 step) process [6]:

1. CO_2_ hydration: $${\text{CO}}_{{2}} + {\text{E}}:{\text{ZnOH}}^{ - } \leftrightarrow {\text{E}}:{\text{ZnHCO}}_{{3}}^{ - } \leftrightarrow {\text{E}}:{\text{ZnH}}_{{2}} {\text{O}} + {\text{HCO}}_{{3}}^{ - } .$$
2. Proton transfer: $${\text{E}}:{\text{ZnH}}_{{2}} {\text{O}} + {\text{B}} \leftrightarrow {\text{H}}^{ + } {\text{E}}:{\text{ZnOH}}^{ - } + {\text{B}} \leftrightarrow {\text{E}}:{\text{ZnOH}}^{ - } + {\text{BH}}^{ + } .$$

His64 is located on the entrance of a cone-shaped active site and its side chain displays two conformations in crystal structures [5, 7, 8]: the inward, pointing toward the zinc (more common in crystal structures at higher pH), and the outward that is pointing toward the solvent (more common in crystal structures at lower pH) [5, 7]. Both conformations interconvert rapidly at 10^7^–10^9^ s^−1^ [9, 10]. Furthermore, it displays a p*K*_a_ value at neutral pH making the proton transfer feasible and efficient at physiological pH. The overall catalytic rate constant (*k*_cat_) in the carbon dioxide to bicarbonate direction increases with higher pH up to a maximum of 1.4 × 10^6^ s^−1^ at pH 9. For the reverse reaction it increases with lower pH up to 6 × 10^5^ s^−1^ at pH 6.5. In contrast, the Michaelis–Menten constant (*K*_M_) is independent from pH [11]. One can think of the catalytic rate constant as (outlined for carbon dioxide hydration): (1) the actual catalytic step, (2) proton transfer to His64, (3) His64 side chain flips to the outward conformation (4) proton transfer to the bulk water. Whatever is rate limiting at the given conditions is reflected in the *k*_cat_.

To date various studies have focused on structural details of the active site, but very few have addressed the electrostatic and dynamic nature of His64. So far only the p*K*_a_ value itself (as the titration midpoints) has been reported by NMR [12] or by indirect methods [13]. What is missing is the actual shape of the pH titration curves for the ionization of His64. Such information would provide information on the mechanism, e.g., a possible energetic coupling to other ionizable groups [14, 15]. Furthermore, structural and dynamic information of His64 at equilibrium in solution using NMR spectroscopy does not exist. NMR spectroscopy has a long history in determining site specific p*K*_a_ values in proteins [15–19]. Recently the kinetic of proton transfer in Asp and Glu side chains could be determined [20]. In addition, NMR spectroscopy allows a high-resolution view on equilibrium, structure and dynamics of proteins in solution.

Here we show that the pH titration curve of His64 can best be described by two p*K*_a_ values of 6.25 and 7.60. The two different p*K*_a_ values of His64 arise from its two conformations, the inward and outward conformations. We show that His64 exists in both conformations at around 1:1 population, independent of pH. In addition, for both conformations the ratio of tautomers in their neutral form is also around 1:1. The life time of each conformation is short and His64 displays high flexibility in both conformations. Finally, we demonstrate that residues close to the active site also sense the protonation and conformational behavior of His64 allowing the assignment of p*K*_a_ values of 6.25 and 7.60 to inward and outward conformations, respectively.

## Materials and methods

### Protein samples

The cDNA of the HCAII wild type (WT) and mutants were codon optimized for expression in *E. coli*, synthesized and cloned into pET-26b (+) (Novagen) using the NdeI/XhoI restriction sites by GenScript (Hong Kong) for expression. The plasmid was transformed into chemically competent *E. coli* TUNER(DE3) cells (Novagen). HCAII was expressed using ModC1 media with ^13^C-glucose instead of glycerol to enable preparation of labeled protein for NMR [21]. At an OD_600_ of 1, protein expression was induced by the addition of isopropyl β-d-1-thiogalactopyranoside (IPTG) to a final concentration of 1 mM. At the same time, the cultures were also supplemented by 1 mMZnSO_4_. After 4 h, the cells were harvested and cell pellets were frozen at -80 °C until processed further.

Frozen cell pellets were thawed and resuspended by in 0.2 M sodium sulfate, 0.1 M Tris–HCl, pH 9.0 and stirred at 4 °C for 16 h with addition of 20 mg of lysozyme and 1 mg DNaseI per 1 L cell pellet. The supernatant from the lysate after centrifugation was directly applied to a *p*-amino-methyl-benzenesulfonamide-agarose resin (pAMBS, Sigma-Aldrich) packed in a disposable gravity column that was pre-equilibrated with 0.2 M sodium sulfate, 0.1 M Tris–HCl, pH 9.0. The column was then washed with 20 column volumes of the same buffer, followed by 20 CV of 0.2 M sodium sulfate, 0.1 M Tris–HCl, pH 7.0. The proteins were eluted from the column with 10 CV of 0.4 M sodium azide, 50 mM Tris–HCl, pH 7.8. Eluted protein was concentrated to 12 mg/mL in Amicon Ultra 15 centrifugal filtration devices (10 kDa MWCO) and dialyzed against 25 mM Tris pH 8. Protein purity was assessed using SDS-PAGE, and the concentration was determined by measuring the UV absorbance at 280 nm.

To prepare apo HCA II, the zinc was removed through rapid dialysis (3 × 30 min) against a chelation buffer (100 mM pyridine-2,6-dicarboxylic acid, 25 mM Tris, pH 7.0). The enzyme was placed in this buffer with 3 changes every 30 min. To remove the chelating agent, the enzyme was buffer exchanged 3 times against 50 mM Tris, pH 7.8. The loss of enzyme esterase activity was verified through activity assays using para-nitrophenylacetate (pNPA), as has been described elsewhere [22]. Following this procedure, it was possible to remove ~ 90% of the zinc from the enzyme, further chelation attempts do not further reduce the esterase activity. For metal substitution, the enzyme was dialyzed against 1 mM CuSO_4_, 50 mM Tris pH 7.8 overnight at 4 °C.

### NMR spectroscopy

For NMR measurements, samples with 0.5–1.0 mM HCAII were dialyzed against 50 mM MES, MOPS, HEPES, TRIS/HCl or CAPS buffer. For assignment, HCAII with Cu^2+^ at the copper center Cu_A_ [23] and apo HCA II [24] were used. For measurements with inhibitor, acetazolamide from a 1 M DMSO stock was added to the sample to achieve an inhibitor concentration of 10 mM. For measuring residual dipolar coupling, a 5% C12E5/hexanol (*r* = 0.96) was used and prepared as described in [25] with the modification that the solution was shaken at 308 K to reach the L_α_ phase. The pH was adjusted with NaOH or HCl on each sample (10% D_2_O) and checked right before the measurement in the NMR tube at room temperature with an inoLab pH 720 pH meter equipped with a Hamilton Spintrode pH electrode.

Unless otherwise stated, the experiments were performed on a Bruker Avance III spectrometer at a static magnetic field of 18.8 T. Titration curves with WT HCAII were measured at 308 K and 314 K. All other measurements were performed at 308 K. pH titrations were monitored by ^1^H^13^C constant time HSQC experiments optimized for the aromatic region with water suppression via gradient selection in a pH range from 5.1 to 9.9. Below this pH additional signals were observed and the dispersion of the spectra was lost at around pH 4.5. pH dependent^13^C *R*_1_ relaxation experiments at His ε1 were acquired with longitudinal-relaxation optimized based relaxation experiments [26]. Residual dipolar couplings (RDC) were measured with aromatic TROSY/AntiTROSY HSQC experiments [26]. All spectra were processed with NMRPipe [27] and analyzed with NMRView [28]. Measured *R*_1_ data were analyzed with PINT [29].

### Assignment

Peak patterns belonging to one amino acid were identified by ^1^H^13^C NOESY-HSQC, TOCSY-HSQC, COSY-HSQC, H(C)N-TROSY and ^1^H^15^N 2J-HSQC experiments. The 5 histidines at the N-terminal were assigned by the HCAII mutants where His was replaced by Ala: H3A, H4A, H10A, H15A and H17A. H36 is the only one which is not influenced by inhibitors, variants e.g., and also shows behavior of a standard solvent exposed histidine. From the remaining histidine residues, only His64 displayed a pH transition and vanishes with Cu^2+^ at the copper center Cu_A_. The rest of the histidine residues display no measurable protonation kinetics. Four of them are in the Nδ1H tautomeric state and one is in the Nε2H state, in agreement with previous reported neutron crystal structures. The three zinc-coordinating histidines show big shifts in apo (metal-free) HCAII, with His119 being in the Nε2H state. The other two can be distinguished by a NOE contact between His96 Hδ2 and His 119Hε1.

His107 Hε1 has an abnormally low chemical shift because it faces the center of the aromatic side chain of Trp209 and makes a hydrogen bond to Tyr194. His122 in the interior of the protein is the remaining one which is also least influenced by the presence of bound inhibitors. For all histidines except His17 a corresponding δ2 peak was found. Based on the chemical shifts from [24] and COSY-HSQC and NOESY-HSQC experiments, all Tyr could be assigned, except Tyr88. Tyr7 was confirmed by the variant Y7F. The patterns of Trp5, Trp16 and Trp209 could be assigned by NOE signals with the neighboring histidines. The assignment was confirmed by RDC measurements and the available assignment of [12] for His64, His107, His119 and His122.

### Data analysis

The pH value was corrected theoretically to the measurement temperature. A random check gave a maximum discrepancy of 0.03 (samples with TRIS/HCl and CAPS are not included due to their mere relevance of the baseline).

The observed chemical shift *δ*_obs_ for a monophasic titration event was fitted to the Henderson–Hasselbalch equation without and with a Hill parameter *n*_H_

$$ \delta_{{{\text{obs}}}} = \frac{{\delta_{{{\text{HA}}}} + \delta_{{{\text{A}}^{ - } }} 10^{{{\text{pH}} - {\text{p}}K_{{\text{a}}} }} }}{{1 + 10^{{{\text{pH}} - {\text{p}}K_{{\text{a}}} }} }}, $$

$$ \delta_{{{\text{obs}}}} = \frac{{\delta_{{{\text{HA}}}} + \delta_{{{\text{A}}^{ - } }} 10^{{n_{{\text{H}}} \left( {{\text{pH}} - {\text{p}}K_{{\text{a}}} } \right)}} }}{{1 + 10^{{n_{{\text{H}}} \left( {{\text{pH}} - {\text{p}}K_{{\text{a}}} } \right)}} }}, $$

whereas *δ*_HA_ and *δ*_A−_ are the chemical shifts of the protonated and deprotonated state, respectively [30]. Multisite titration curves were fitted to

$$ \delta_{{{\text{obs}}}} = \mathop \sum \limits_{i} \frac{{{\updelta }_{{{\text{HA}}}}^{i} + \delta_{{{\text{A}}^{ - } }}^{i} 10^{{{\text{pH}} - {\text{p}}K_{{{\text{a}},i}} }} }}{{1 + 10^{{{\text{pH}} - {\text{p}}K_{{{\text{a}},i}} }} }}, $$

where *i* represents the respective site. In case of a Coulomb coupled system, p*K*_a,i_ describes the net protonation (macroscopic behavior) [14] and contains for two-site model two microscopic, p*K*_a_ values and one coupling parameter which describes the Coulomb interaction. In case of one titration site with two conformations, latter parameter describes the conformation change.

Accordingly, the pH titration curves of His64 and its surrounding were fitted with a nonlinear least-squares regression analysis (Levenberg–Marquardt algorithm) to standard Hendersson-Hasselbalch equations with 2 macroscopic p*K*_a_ values in a global way using Matlab. The pH titration curves of His64 were also fitted to a monophasic transition and to the Hill model. For error estimation, Monte-Carlo simulations with 1000 steps, a random variation of pH (± 0.1), ^1^H(± 0.01 ppm) and ^13^C (± 0.02 ppm) chemical shift were executed. The latter was determined by analyzing the chemical shifts of peaks without any transition (standard deviation of ± 0.003 for ^1^H and ± 0.012 for ^13^C).

For the TROSY/AntiTROSY experiments a measurement uncertainty of 2 Hz was assumed. The titration curve of the *J*-coupling was fitted in the same way mentioned above and was subtracted from the data measured with alignment medium. The calculated RDCs were analyzed using REDCAT [31] within the NMR virtual environment [32]. Based on the RDCs of ^1^Hε1–^13^Cε1 at His17, His94, His96, His107, His119 and His122, the order parameters were calculated and the RDC for His64 was back-calculated for different high-resolution crystal structures (PDB ID: 6luw, 2ili, 3ks3, 5yuj, 1tbt, 4y0j, 1ca3 and 1te3).

## Results and discussion

**Fig. 1:**
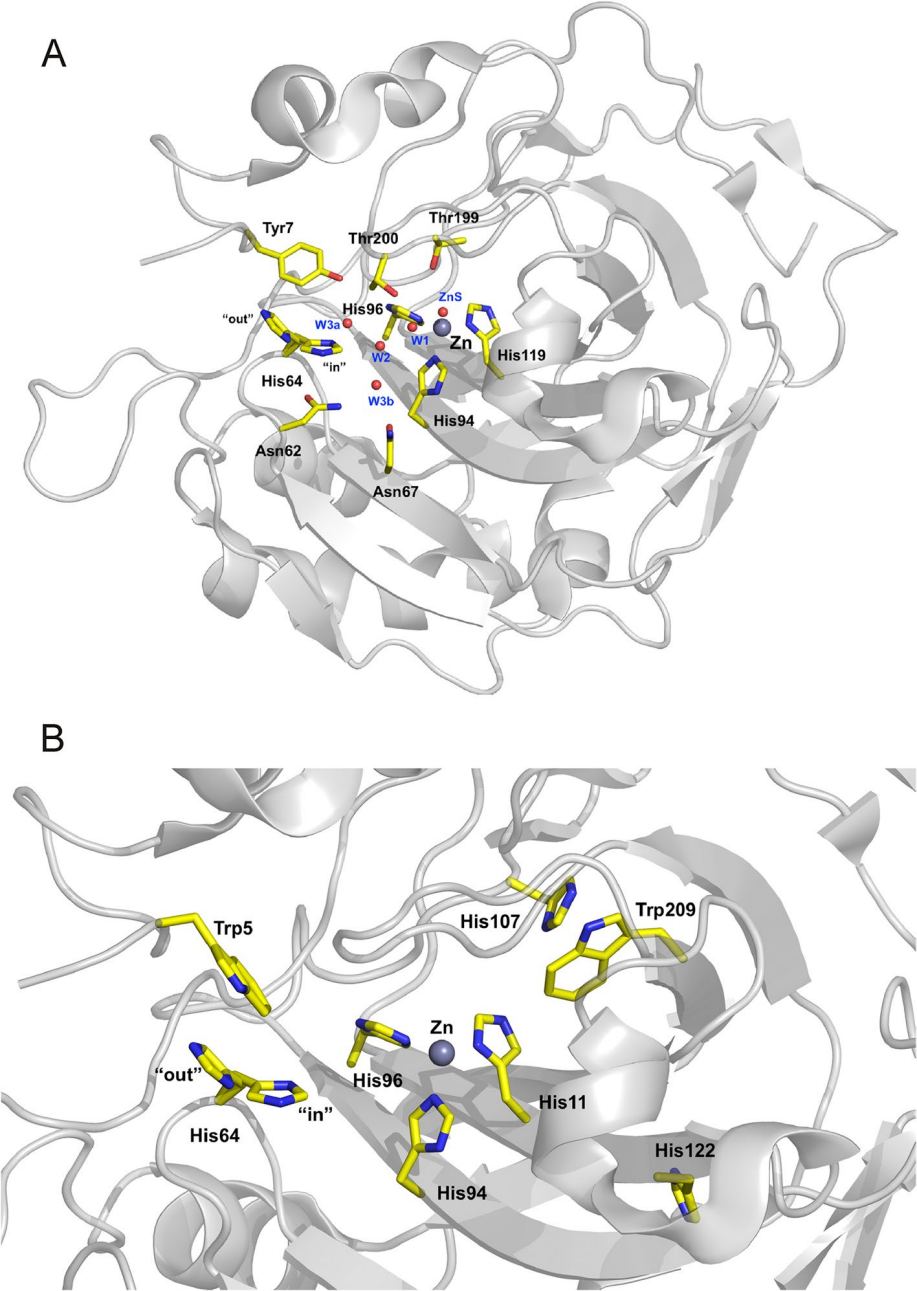
Active site (**A**) and important residues used in this study (**B**) of wild type HCA II based on PDB ID2ili [40]. Residues are shown in stick form and are as labeled, the zinc ion is shown as a black sphere and water molecules as red spheres. Figure was prepared using PyMOL [41]

The active site of carbonic anhydrase II (Fig. 1) consists of a zinc ion that is tetrahedrally coordinated by three histidine residues (His94, His96, His119) and the catalytic zinc-bound solvent. The catalytic solvent can be either a water or hydroxide molecule, depending on the reaction direction of catalyzed by carbonic anhydrase. In the direction toward CO_2_-hydration direction, Zn-OH^−^ is connected to His64 via a hydrogen-bonded water network. His64 is observed to occupy both inward and outward conformations in crystal structures and this feature is thought to be consistent with its function of shuttling protons between the active site and the bulk solvent. In its outward conformation in crystal structures His64 makes pi-stacking contacts with Trp5 and is in proximity to Tyr7 (Fig. 1). In the aromatic ^1^H^13^C HSQC spectra we see all expected 12 signals from Hisε1 (Supplementary Fig. S1): His64, the zinc ligands (His94, His96, His119), His107 which is located adjacent to zinc ligands, His122 which is buried in the hydrophobic core, the solvent accessible His36, and the 5 His residues found close to the N-terminus (His3, His4, His10, His15, His17). Initial assignments are based on [12], and were further augmented by NOEs, mutations, Cu^2+^ and inhibitor binding, as well as the pH dependence of the signals. In addition, signals from Trp5, Trp16 and Trp209 could be identified (ε3, ζ3, η2 and ζ2) and assigned by NOEs and inhibitor binding. All Tyr residues, except Tyr88, could be observed and assigned confidently using the previous assignment from [24]. The assignment of Tyr7 was additionally confirmed by mutation to phenylalanine (Y7F).

### The pH titration curve of His64 can be described best by two p***K***_a_ values.

**Fig. 2:**
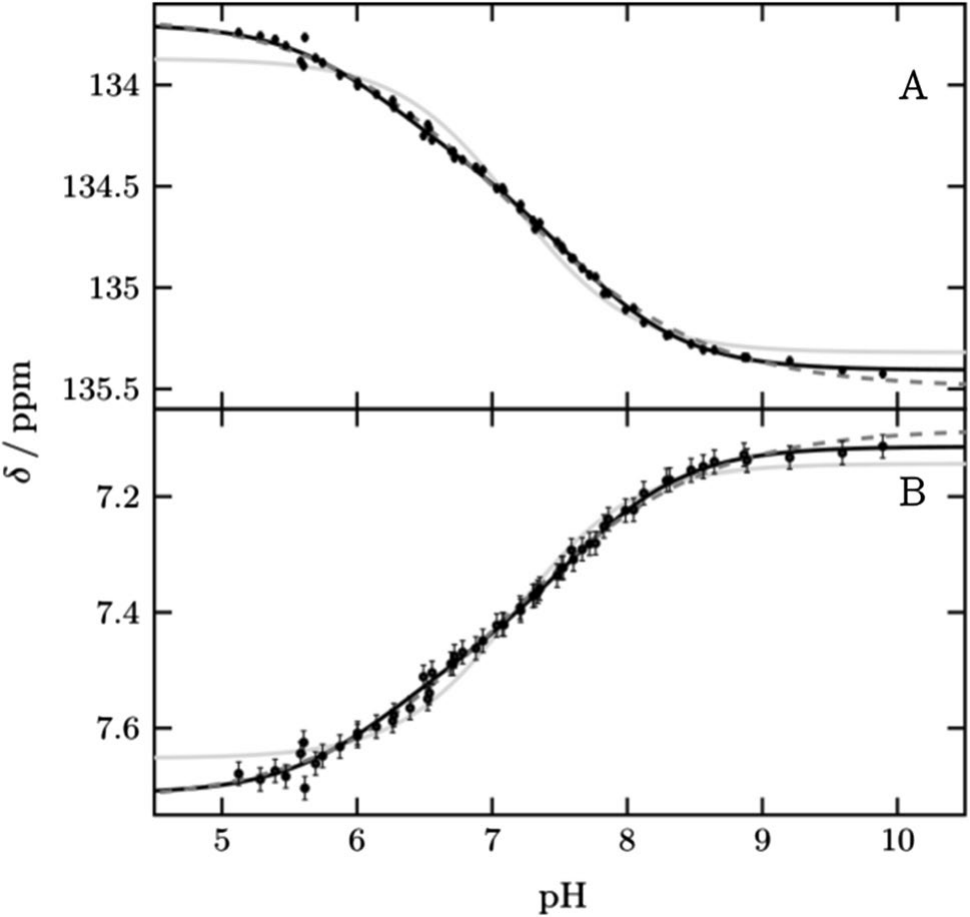
pH titration curves of His64 (black points) for (**A**) ^13^Cε1 and (**B**) ^1^Hε1 fitted globally to two p*K*_a_ values (black line), one p*K*_a_ value and hill coefficient (black dash) and one p*K*_a_ value (gray line). Results are two macroscopic p*K*_a_ values 6.20 ± 0.13 and 7.62 ± 0.07 for the biphasic transition, a p*K*_a_ value of 7.12 ± 0.04 and *n*_h_ 0.60 ± 0.03 for the hill model and a p*K*_a_ value of 7.17 ± 0.03. For the macroscopic model, a global fit with the affected vicinity was also performed to improve the accuracy to 6.25 ± 0.08 and 7.60 ± 0.06. These curves are shown in Fig. S2

We monitored His64 ε1 between pH 5.1 and pH 9.9 over a total of 58 points (Fig. 2 and Supplementary Fig. S2). The protein is well folded over this pH range, including at the theoretical p*I* of 6.9, in agreement with previous studies [5, 24]. Below pH 5.1 we observed a change for active site and inner histidines (Supplementary Fig. S3). The transitions of ^1^Hε1 and ^13^Cε1 are broadened and cannot be described by one p*K*_a_ value alone. Such broadening of pH transitions arises from energetic coupling of two or more ionizable groups. By using two macroscopic p*K*_a_ values [14, 15] the data can be described well, resulting in two values, p*K*_a1_ of 6.25 ± 0.08 and p*K*_a2_ of 7.60 ± 0.06 with relative amplitudes of 40 and 60%, respectively. Alternatively, the data can be described by one *pK*_a_ value and negative cooperativity, implemented by a cooperativity factor (the so-called Hill coefficient) below one. This approach results in a p*K*_a_ value of 7.12 ± 0.04 and a Hill coefficient of 0.58 ± 0.03. This p*K*_a_ value agrees with the midpoint of the transition reported so far. From now on we are using the macroscopic model with two p*K*_a_ values to describe broadened transitions as it provides improved granularity and explains the behavior better. However, in principle the same conclusions of the following chapters can be drawn if broadened transitions are described by the Hill model.

### The two p***K***_a_ values of His64 arise from its outward and inward conformations and not from Coulomb coupling

The observed broadened pH transition can be explained by two different scenarios. The first scenario is that His64 is energetically coupled with another ionizable group that is in close proximity. There are three potential groups that could be responsible for coulomb coupling: (1) the zinc-bound solvent (H_2_O or OH^−^), (2) Tyr7, and (3) N-terminal histidines, His4 presumably being the closest, although often not resolved in crystal structures due to N-terminus disorder. In these cases, the proton occupancy of His64 and the other ionizable group would depend on each other with added protons shared between the two groups. The two macroscopic p*K*_a_ values would describe the two protonation events of the total system and represent the p*K*_a_ value of His64 and the other ionizable group.

Another scenario is that the two macroscopic p*K*_a_ values are the p*K*_a_ values of His64 in its inward and outward conformation (4). Since these two conformations put the His64 side chain in two different chemical surroundings, it is likely that His64 displays two different p*K*_a_ values. In fact, it would be a coincidence if p*K*_a_ values for the inward and outward conformations were identical. All these possibilities would lead to new important findings: (1) His64 and the zinc-bound water, i.e., the proton shuttle and the catalytic group, are one energetic unit when it comes to protons, despite their separation of three water molecules as observed in crystal structures; (2) His64 and Tyr7 are one energetic unit, connecting a possible alternate proton transport pathway; (3) His64 and the N-terminal His would be an energetic unit, suggesting a further coordinated proton transport; (4) The shuttling process of His64 not only results from structural differences but also from energetic differences.

**Fig. 3:**
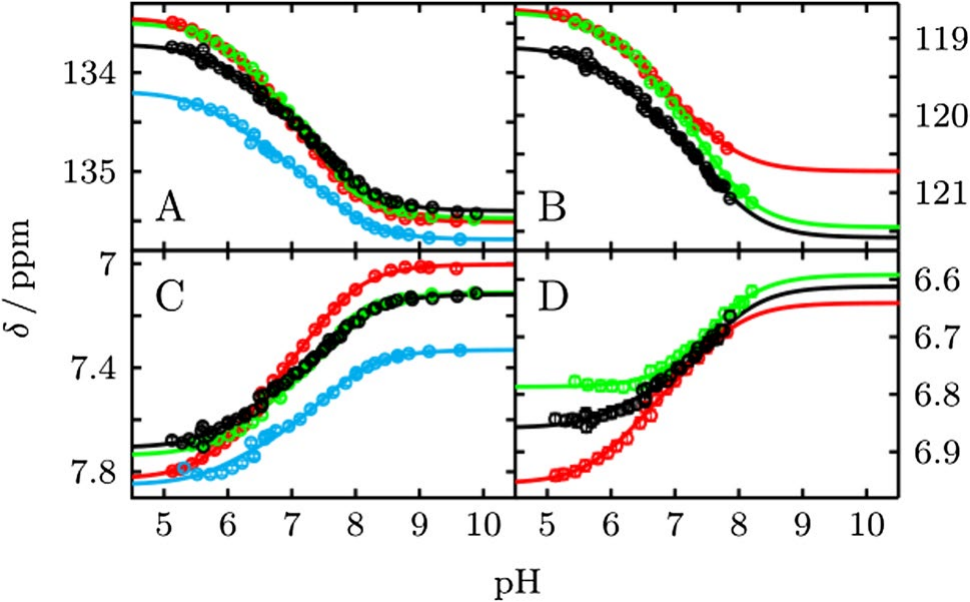
pH titration curves of His64 in different variants for **A** ^13^Cε1, **B** ^13^Cδ2, **C** ^1^Hε1 and **D** ^1^Hδ2 fitted globally to two p*K*_a_ values. wt is shown in black, H4A in green, Y7F in blue and wt with the inhibitor acetazolamide bound in red. Results are summarized in Table 1

To investigate case (1) we repeated the pH titration experiments of His64 in the presence of the inhibitor acetazolamide, which displaces the zinc-bound water. For case (2) we replaced Tyr7 by a Phe residue, and for case (3) we replaced His4 by Ala. In all three cases we do not observe any differences in the pH profiles of His64 (Fig. 3 and Supplementary Fig. S2), the transition remains broadened, with p*K*_a_ values shown in Table 1. By ruling out the other possibilities we conclude that the pH transition of His64 reports on the two different p*K*_a_ values of His64, corresponding to the "in" and "out" conformation. However, from these experimental data it is not definitively possible to assign the two p*K*_a_ values to these conformations. We will discuss the most likely assignment of the p*K*_a_ values later in the manuscript in more detail. What can be concluded at this point is, that His64 is in constant motion between the two conformations, so that one NMR signal for His64 ε1 gives rise to two p*K*_a_ values. His64 ^13^Cδ2, a chemical shift which is diagnostic of the tautomeric state of a histidine in its neutral form (HNε2 or HNδ1), displays exactly the same broadened transition curve (Fig. 3B). It comes to a chemical shift of around 121 ppm, indicating roughly a 50/50 distribution of both tautomeres, in agreement with earlier observations on ^15^Nε2 and ^15^Nδ1 [12]. Since the transition is broadened exactly the same way for ε1 and δ1, we further conclude that both conformations of His64 exist in a 50/50 distribution of tautomers (Nε2H or Nδ1H) and that neither conformation is 100% in one tautomer nor is the other conformation 100% in the other tautomer.

**Table 1:** Determined macroscopic p*K*_*a*_ values and the corresponding difference in chemical shift (Δδ) of ^13^Cε1 and ^13^Cδ2 position of His64 for various pH titrations at 308 K

	p*K*_*a*,1_	p*K*_*a*,2_	Δδ_1_(Cε1)/ppm^a^	Δδ_2_(Cε1)/ppm^a^	Δδ_1_(Cδ2)/ppm^a^	Δδ_2_(Cδ2)/ppm^a^
WT^b^	6.25 ± 0.08	7.60 ± 0.06	0.67 ± 0.06	1.02 ± 0.06	0.97 ± 0.09	1.50 ± 0.14
WT (313 K)^b^	6.05 ± 0.12	7.41 ± 0.07	0.68 ± 0.08	1.03 ± 0.07	0.91 ± 0.11	1.55 ± 0.13
WT + acetazolamide	6.27 ± 0.17	7.43 ± 0.12	0.86 ± 0.20	1.20 ± 0.21	1.06 ± 0.21	1.04 ± 0.26
H4A	6.34 ± 0.12	7.53 ± 0.09	0.80 ± 0.13	1.18 ± 0.13	0.98 ± 0.19	1.80 ± 0.19
Y7F	6.13 ± 0.13	7.54 ± 0.05	0.64 ± 0.06	0.86 ± 0.07		

^a^In all cases and for both, Cε1 and Cδ2, the ratio of chemical shift difference between p*K*_*a*,1_ and p*K*_*a*,2_ is about 40%/60%.

^b^The temperature dependence for His64 between wt at 308 K and 313 K corresponds to the expected behavior [39].

^c^Results were estimated by fitting the positions of His64 and its surrounding in a global way. More details can be seen in Supplementary Fig. S2. Reported errors are from Monte-Carlo simulations

### His 64 exists in multiple conformations independent from pH

**Fig. 4:**
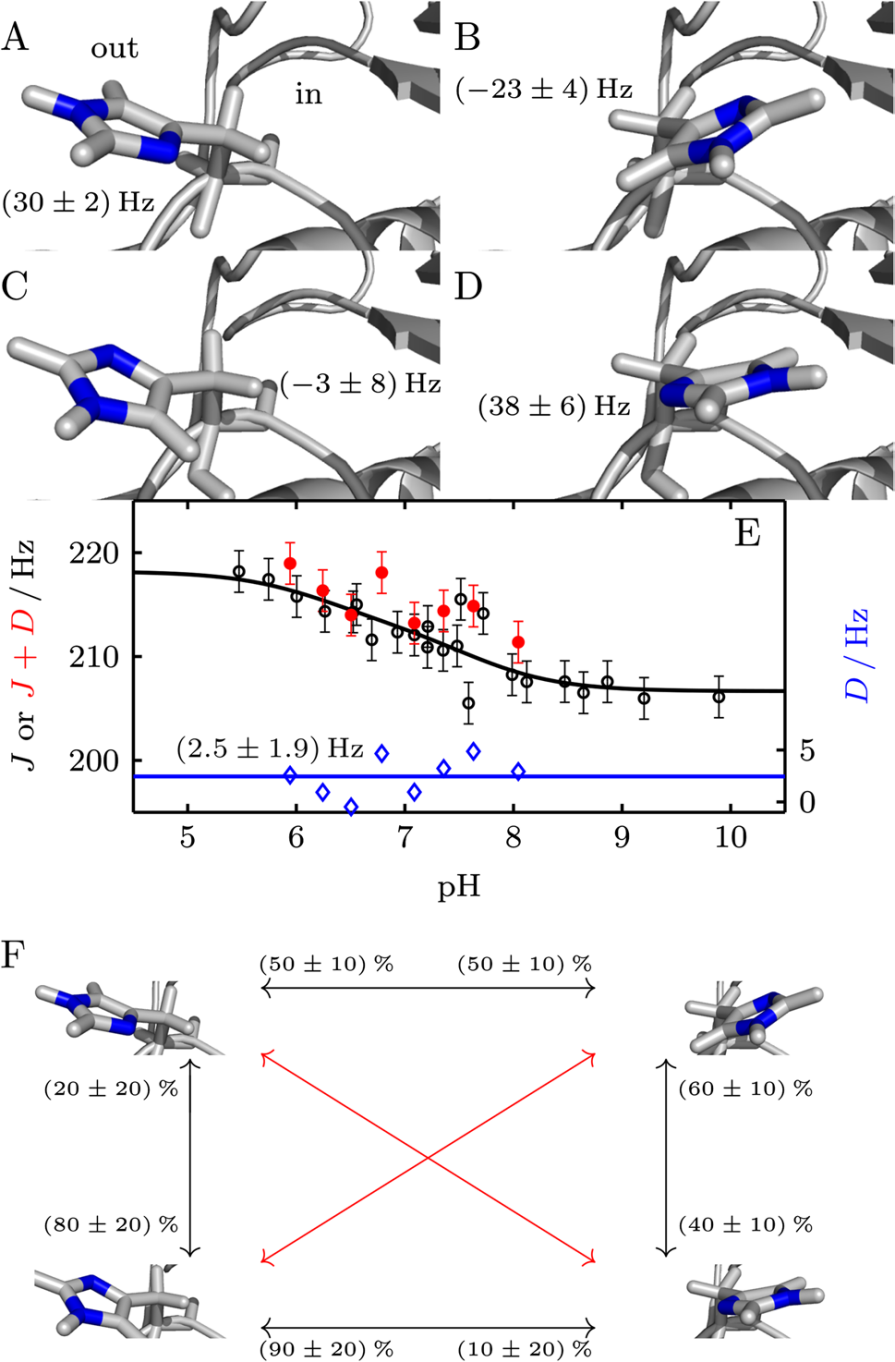
Calculated RDCs for different conformations of His64 and measured values. **A** Shows His64 in the inside and **B** in the outside conformation, whereas Nδ1 is directed inward (pdb-id 2ili). For **C**, **D**, Nδ1 is directed outward (PDB ID 1tbt). RDCs were calculated from different structures (pdb-id 6luw, 2ili, 3ks3, 5yuj, 1tbt, 4y0j, 1ca3 and 1te3) and the mean and the mean deviation are specified. In **E**, measured ^1^*J*^1^_He1–_^13^_Ce1_-coupling values (black circles) for wt were fitted to the two macroscopic p*K*_a_ values of 6.25 and 7.60 (black line). The measured *J*-coupling in combination with the residual dipolar coupling *D* for wt with alignment medium (5% C12E5/hexanol (*r* = 0.96), prepared as described in [25] with the modification that the solution was shaken at 308 K to reach the L_α_ phase) are shown in red. For the solely residual dipolar coupling, the fitted *J*-coupling was subtracted from the measurement points (blue). A mean residual dipolar coupling of (2.5 ± 1.9) Hz was obtained. **F** shows examples of two-state distribution of His64, that would fulfill the experimental RDC. Two-state combinations that are not possible are indicated with red arrows. Three- or four-state combinations are possible and likely, but their quantification is not meaningful

The His64 side chain has been found to coexist in two distinct conformations in many crystal structures [5, 7]: the so-called in and out. Furthermore, both conformations are divided further into aromatic rings that are rotated about 180° around chi-2 (defined as the angle in Cα-Cβ-Cγ-Cδ) (Fig. 4A–D). Since the outward conformation seems more favored in crystals at lower pH, and the inward conformation more in crystals at higher pH, it has been suggested that the distribution follows a pH dependence. Observing only one set of NMR signals for His64 and detecting two p*K*_a_ values for "in" and "out" indicates a fast transition between the two conformations at equilibrium. We were unable to structurally characterize the conformations of His64 by NOE. A possible explanation for this is, that the residence time of His64 in a distinct conformation is too short for a buildup of the NOE. In order to get structural insight into the orientation of the aromatic side chain of His64 in the solution ensemble and its pH dependence we acquired RDCs of His64 ε1 at different pH values. The *J*-coupling of ^1^Hε1-^13^Cε1 undergoes changes in the expected pH dependent manner, derived RDCs are small (2.5 ± 1.9 Hz) and do not change with pH (Fig. 4E). We calculated RDCs of His64 ε1 from different high-resolution crystal structures, that cover different orientations of His64, and experimental alignment tensors determined from static histidines located in the interior of the protein (Fig. 4A–D and Supplementary Fig. S4). The results show that a single conformation of His64 cannot explain the experimental RDC. Therefore, His64 exists in an equilibrium of more than one conformation, "in" and "out" and/or different orientations of the ring around chi-2. Possible two-state combinations that fulfill the experimental data are shown in Fig. 4F. Three- or four-state combinations are possible and likely, but their quantification is not meaningful. Based on the observation that we do not observe significant line broadening or measurable NOEs, one can estimate that these motions are at least 10^5^ s^−1^, and likely higher since the maximal catalytic rate constant is 1.4 × 10^6^ s^−1^. Furthermore, a change in RDCs with pH was not observed. A pH dependent substantial change in the "in" and "out" equilibrium, however, would result in a change of experimental RDCs. Taken together our results show, that the side chain of His64 in solution exists in multiple conformations (with a favored distribution between "in" and "out") and that the equilibrium is not affected by pH, within experimental errors. From this follows that the observed changes in enzymatic activity by pH do not arise from structural changes in the proton shuttle, but only from pH altered protonation/deprotonation rate constants. This is further demonstrating the very efficient catalysis of carbonic anhydrase, which is only limited by proton transfer events.

### His64 is unrestricted independent from pH

**Fig. 5:**
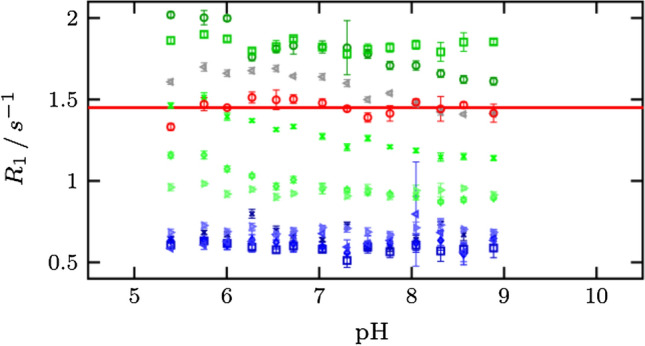
Measured *R*_1_ values at the ^13^Cε1 position for all 12 histidines: His64 (red), the inner histidines His94, His96, His119, His107 and His122 (from dark blue to light blue), His3, His4, His10, His15 and His17 located at the N-terminus (from dark green to light green) and H36 (gray)

In order to investigate the dynamic nature of His64 further, we acquired ^13^Cε1 *R*_1_ rate constants for all histidine side chains over a range of pH. *R*_1_ is only sensitive to dynamics below the overall correlation time of the protein, which can be estimated for carbonic anhydrase under given conditions to be 10 ns or less [33, 34]. This translates to processes over 10^8^ s^−1^, which is above the maximal catalytic rate constant of 1.4 × 10^6^ s^−1^. In other words, fluctuations within the "in" and "out" conformations, and/or the actual movement of going between "in" and "out" has to be extremely high. Computational studies find that His64 interconverts between the two conformations at rates of 10^7^–10^9^ s^−1^ [9, 10]. This makes it likely that *R*_1_ is reporting on motions between "in" and "out". All ^13^Cε1 *R*_1_ rate constants for all His residues, including His64 (with exception of His10 and His36) appear to be independent from pH. Therefore, there is no pH induced change in the fast dynamics of these side chains (Fig. 5). As can be expected from their structure and role, the zinc ligands His94, His96, and His119, and His107 and His122, which are buried in the interior of the protein, display low ^13^Cε1 *R*_1_ rate constants in the 0.6 to 0.7 s^−1^ range, reflecting their restrictive environment. In contrast, His3 and His4 display high ^13^Cε1 *R*_1_ rate constants of around 1.8 s^−1^, indicating their unrestricted nature, also in agreement with their relative disorder in crystal structures. His15 and His17 show moderate ^13^Cε1 *R*_1_ rate constants of around 1 s^−1^, in agreement with their well-resolved structures. ^13^Cε1 *R*_1_ rate constants for His10 and His36 are in the range of 1.2 to 1.6 s^−1^. The protein shuttle His64 displays high ^13^Cε1 *R*_1_ rate constants of 1.5 s^−1^, showing it is way more unrestricted than the zinc ligand and inside His residues and even more so than some of the N-terminal His. Therefore, His64 in the solution ensemble is less restricted and defined as one would think based on crystal structures alone. The collection of crystal structures that capture His64 in the "in" and "out" conformations and with different chi-2 orientations seems to be a better description of the system. Our results show that His64 is never static or structurally restricted and always ready to change conformation. In other words, His64 can be seen to be frustrated [35–37] in all its states, independent from pH. This might be a prerequisite for its role as an efficient proton shuttle. Furthermore, it supports the findings that it can change conformation very quickly and is not influenced by pH or any other forces.

### Protonation behavior sensed by residues in the active site

Information about the protonation behavior of the zinc-bound water/hydroxide was obtained by measuring pH titration curves of residues in closest proximity to the zinc-bound water, namely His96, His107, His119 and Trp209. None of these residues are titrating by themselves in the studied pH range, which was experimentally confirmed. The zinc-coordinating histidines do not display the typical large changes in amplitudes during pH titration. It is not expected that they change their protonation state since the free electron pairs on the imidazole nitrogens are directly involved in coordination of the zinc. The only titrating groups in the active site are His64 (in both conformations) and the zinc-bound water. His64 is ~ 7.5 Å [38] away from the zinc-bound water, connected via a hydrogen-bonded water network. As outlined above, the zinc bound water is not influencing the pH profile of His64.

**Fig. 6:**
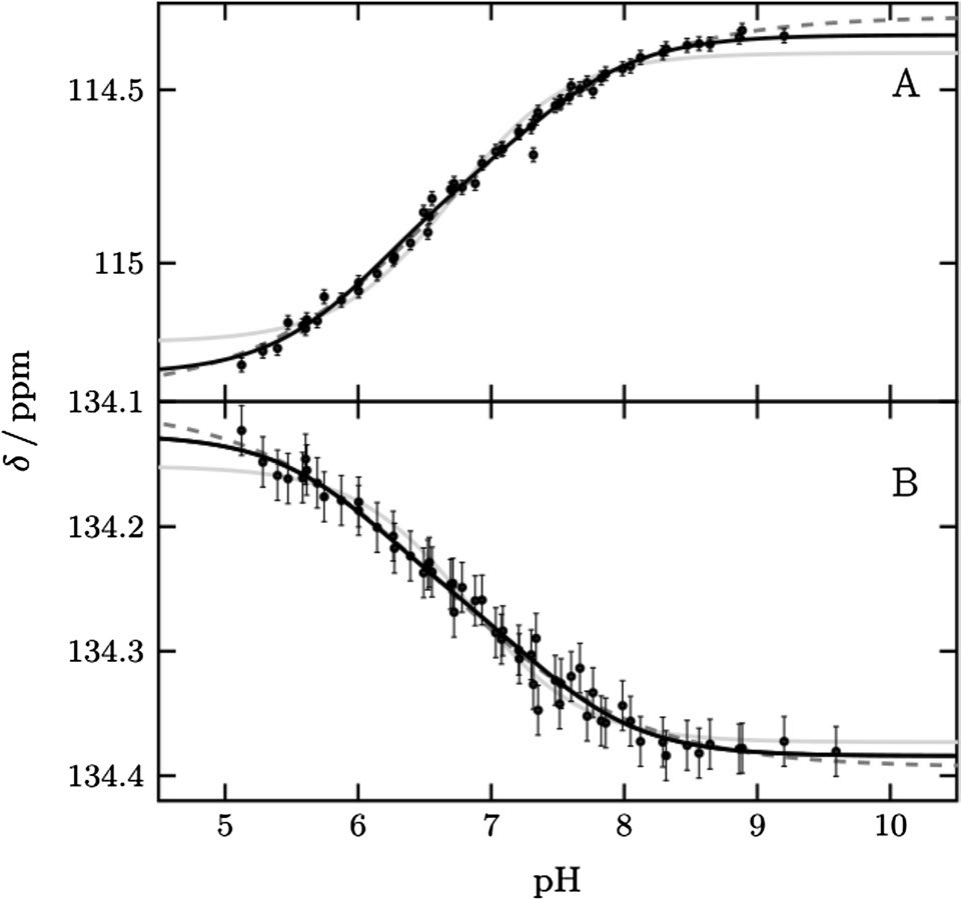
pH titration curves of inner histidines (black points) for **A** His119 ^13^Cδ2 and **B** His107 ^13^Cε1 fitted to two p*K*_a_ values (black line), one p*K*_a_ value and hill coefficient (black dash) and one p*K*_a_ value (gray line). Results are two macroscopic p*K*_a_ values of 6.11 ± 0.12 and 7.37 ± 0.08 for the biphasic transition and a p*K*_a_ value of 6.65 ± 0.05 and *n*_h_ 0.60 ± 0.04 for the hill model. For a monophasic transition of solely His119 ^13^Cδ2 and His107 ^13^Cε1, it results in a p*K*_a_ values of 6.71 ± 0.04 and 6.83 ± 0.09, respectively. For the macroscopic model and the hill model, a global fit with the inner histidines and W209 was performed

The pH profiles acquired on the active site residues are broadened (Fig. 6), and two p*K*_a_ values (6.11 ± 0.12 and 7.37 ± 0.08) or a Hill coefficient (0.60 ± 0.04 with a p*K*_a_ value of 6.65 ± 0.05) is required to describe them well. This means that they sense at least two of the three possible titration events of the zinc-bound water, His64 "in" and His64 "out". The two p*K*_a_ values determined by the pH titration of inside residues are very similar to the two p*K*_a_ values of His64 (6.25 ± 0.08 and 7.60 ± 0.06, respectively). The expected impact of the three possible titration events on the chemical shifts of the studied active site residues, based on distance, is: zinc-bound water > His64 "in" > His64 "out". In order to measure the impact of the supposed p*K*_a_ value of the zinc-bound water (6.8–7.0) we analyzed the pH profiles with two or three fixed p*K*_a_ values: (i) the two p*K*_a_ values of His64 as fixed values from the His64 titration, (ii–iii) one of the His64 p*K*_a_ values fixed together with supposed p*K*_a_ value of the zinc-bound water, (iv) as well as three p*K*_a_ values (two from His64 and the supposed zinc-bound water). Only (i) and (iv) are a good fit to the data. *F*-statistics (Supplementary Table S1) further elaborate that case (i) and (iv) are the best descriptions of the data, but it is not possible to distinguish between these two cases. However, it should be noted that the amplitude (change in chemical shift) for the p*K*_a_ value of the zinc-bound water is very low, in contrast to considerations stated above.

**Fig. 7:**
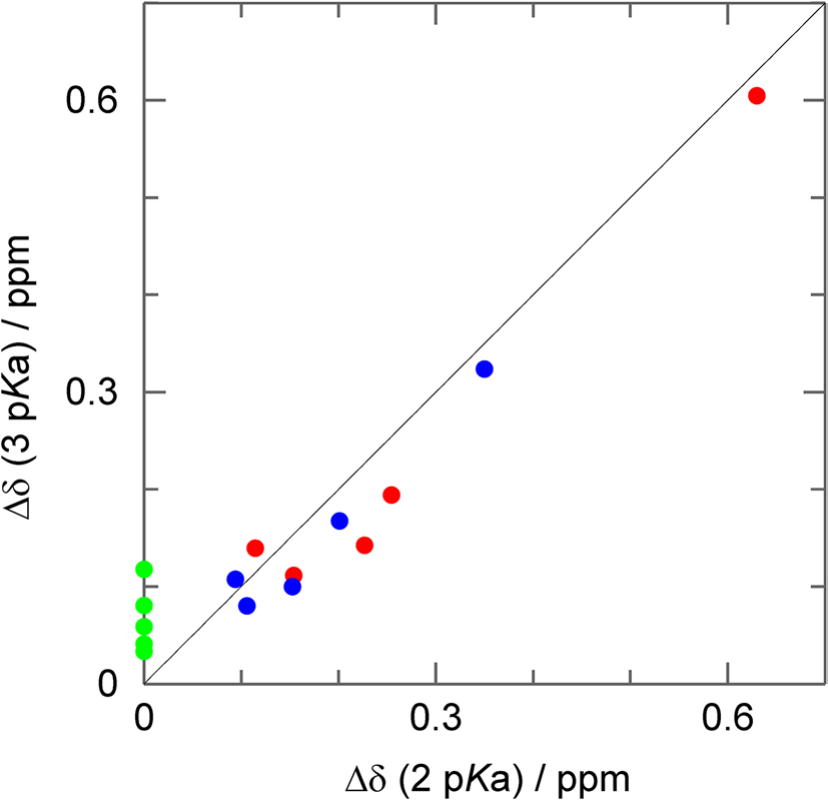
Correlation of** c**hemical shift differences Δδ of the different transitions of residues surrounding the zinc-bound water for the 3 p*K*_a_ versus the 2 p*K*_a_ approach. Values for p*K*_a_ 6.25 are shown in red, for p*K*_a_ 7.60 in blue and for p*K*_a_ 6.80 (which is only used in the 3 p*K*_a_ approach) in green. Theoretical ideal correlation is shown as a black line

By substituting the zinc-bound hydroxide/water with the inhibitor acetazolamide, the protonation profile of the inside residues changes significantly. Here, there is no zinc-bound water anymore, and the water network to His64 is interrupted, which also impacts how His64 is sensed. In total there are three possibilities to explain our data (summarized in Fig. 7). The pH profile is caused only by the two His64 p*K*_a_ values alone. This is the simplest approach to describe the experimental data. Since the amplitude for the lower p*K*_a_ value measured on the inside residues is larger than those measured on His64, this would assign the lower p*K*_a_ value to the His64 inward conformation and the higher p*K*_a_ value to the His64 outward conformation. In this simplest explanation of the data the zinc-bound water does not contribute to chemical shift changes. In the second approach, the findings are very similar to the first approach. The only difference is that a third p*K*_a_ value of 6.8 is additionally fitted to the data, which represents the zinc-bound water. The least likely explanation, which we only mention for the sake of completion, would be that the zinc-bound water titrates at the lower p*K*_a_ value of His64 by chance and the higher p*K*_a_ value (with the lower amplitude) arises from His64 inward conformation.

In summary, we can confidently assign the two p*K*_a_ value of 6.25 to the inside conformation of His64 and p*K*_a_ value of 7.60 to the outside conformation of His 64. The zinc-activated water either does not titrate independently of His64, or at a p*K*_a_ value of around 6.8 and gives rise to a surprisingly small amplitude in the surrounding residues.

### Connections between His64 and enzymatic activity

Carbonic anhydrase is an efficient enzyme with a high catalytic rate constant (*k*_cat_) catalyzing the reversible hydration of carbon dioxide. Both rate constants for the forward and reverse reactions show pH dependence. The rate constant in the carbon dioxide to bicarbonate (with production of a H^+^ that has to be shuttled out) direction increases with higher pH (up to pH 9), while for the reverse reaction (addition of a H^+^ that has to be shuttled in) it increases with lower pH (up to pH 6.5). The reaction toward bicarbonate is extensively studied, with a derived p*K*_a_ value of the activity around 6.8–7.0. It is often attributed to the p*K*_a_ value of the zinc-bound water [6]. However, there is no direct match to the here derived p*K*_a_ values of His64: 6.25 (inward conformation), 7.60 (outward conformation), 7.12 (combined p*K*_a_ values from the Hill model). Therefore, the activity possibly depends on both p*K*_a_ values of His64 in the "in" and "out" conformation. Here we want to discuss our findings in regard to these established principles.

The side chain of His64 occupies at least two conformations in solution and in crystal structures, the inward and outward conformation, with roughly 1:1 occupancy, high flexibility and short life times. Life times can by estimated to < 10 µs (RDCs), < 0.7 µs (enzymatic turnover) or even < 10 ns (*R*_1_). The *R*_1_ based estimation seems to be in good agreement with computational studies [9, 10]. Based on our measurements, these structural and dynamic behaviors of His64 are pH independent. These findings are in full agreement with high enzymatic activity in both directions. While the observed dynamic nature of His64 may be a prerequisite for the high enzymatic activity, it is likely not connected to the pH dependence of the activity.

The possible pH dependent processes that affect the enzymatic activity are therefore the proton transfer between the active site (zinc-bound water and water network) and His64, and the transfer of the proton between His64 and the bulk water. These processes are directly linked to the two p*K*_a_ values determined in this study. Two different p*K*_a_ values of His64 for its two conformations would be an excellent explanation for one efficient enzyme reaction but fails to explain the efficient reverse reaction (bicarbonate dehydration). However, if the conformational change in His64 is fast enough in both directions, His64 in both conformations will share protonation properties of both p*K*_a_ values. In other words, the continuous change in the conformations of His64 with short life time enables efficiency in both directions.

The combination of the two different p*K*_a_ values of His64 results in a pH profile that qualitatively matches the profile of activity. Since the intrinsic p*K*_a_ value of the zinc-bound water is not known directly, there are three possible ways how the pH profile of enzymatic activity is created. Firstly, the protonation state of the zinc-bound water is independent from pH. We have not seen any substantial changes of the chemical shifts of residues in close proximity of the zinc-bound water other than those arising from the two p*K*_a_ values of His64. Since the zinc-bound water is isolated deeper inside the protein, it is conceivable that it doesn't interact with the bulk solvent and the ratio of hydroxide and water is fixed and independent from pH. In this case the pH profile of enzymatic activity arises only from His64. This is in agreement with findings that proton transfer (between the zinc-bound water and His64, and/or between His64 and the bulk water) is rate limiting. Secondly, the p*K*_a_ value of the zinc-bound water is undetectable by NMR titrations for unknown reasons, but it is close to the p*K*_a_ value of enzymatic activity and the combined p*K*_a_ value of His64. Here His64 and the zinc-bound water would display excellent p*K*_a_ matching and both are potentially responsible for the pH profile of the enzymatic activity. Thirdly, the p*K*_a_ value of the zinc-bound water matches the p*K*_a_ value of His64 in its outward conformation. Thereby the NMR pH titration curves monitored on inside residues and the pH profile of enzymatic activity arise from a combination of the zinc-bound water and His64 in its inward conformation. Independent from these three cases, the p*K*_a_ values of His64 are directly responsible for the activity profile.

It should be noted that our results are also in agreement with the model of Shimahara et al*.* [12]. Here, the proton shuttling isn’t explained primarily by the flip of His64, but by a mechanism with all three states of the histidine, the positive charged one and the two neutral tautomeric states. As Shimahara already observed for the overall His64, we also have determined the ratio of the two neutral tautomers as being equal for both the inward and the outward conformers. In addition, we have established that His64 exists in both conformations independent from pH and undergoes continuous transitions between them. This provides a motivation to extend the Shimahara model from a static to a more dynamic one. His64 with two different p*K*_a_ corresponding to its two different conformers nominally results both in good proton donor and proton acceptor characteristics for the zinc-bound water as well as the bulk water. It is also possible that for both models the shuttling of protons via conformational exchange and proton transfer via the tautomeric states go hand in hand.

## Conclusions

His64, the presumed proton shuttle of human carbonic anhydrase II, coexists in two conformations in solution: the inward and outward conformations. The ratio of these conformations is roughly 1:1, as determined from NMR titrations and RDC experiments and does not, or perhaps only very slightly, change with pH. His64 displays two p*K*_a_ values, 6.25 for "in" and 7.60 for "out." The ratio of tautomeres in its neutral form is also roughly 1:1 for both conformations. The two conformations interconvert rapidly and are highly flexible on the ps to ns time scale, confirming His64 is never static but instead always ready to change conformation. These findings built an energetic, dynamic and solution ensemble-based framework for the high enzymatic activity of carbonic anhydrase II.

### Supplementary Information

Below is the link to the electronic supplementary material.

Supplementary file1 (DOCX 1451 KB)

## Data Availability

All data generated or analyzed during this study are included in this published article and its supplementary information files.
