# Supplementary material for: Energetics and dynamics of the proton shuttle of carbonic anhydrase II
Source: Cell Mol Life Sci. 2023 Sep 9;80(10):286. doi: 10.1007/s00018-023-04936-z (PMC10492700; doi:10.1007/s00018-023-04936-z)
Supplement: Supplementary file 1 — Supplementary file1 (DOCX 1451 KB) [file 18_2023_4936_MOESM1_ESM.docx]

Supporting Information

**Energetics and Dynamics of the Proton Shuttle of Carbonic Anhydrase II**

Heiner N. Raum^1^, Suzanne Zoë Fisher^2,3^ and Ulrich Weininger^1*^

^1^ Institute of Physics, Biophysics, Martin-Luther-University Halle-Wittenberg, D-06120 Halle (Saale), Germany

^2^ Department of Biology & Lund Protein Production Platform, Lund University, Sölvegatan 35, SE_22362 Lund, Sweden.

^3^ Scientific Activities Division, European Spallation Source ERIC, P.O. Box 176, SE-22100 Lund, Sweden

* Correspondence:

email: ulrich.weininger@physik.uni-halle.de

phone: +49 345 55 28555

fax: +49 345 55 27161

**
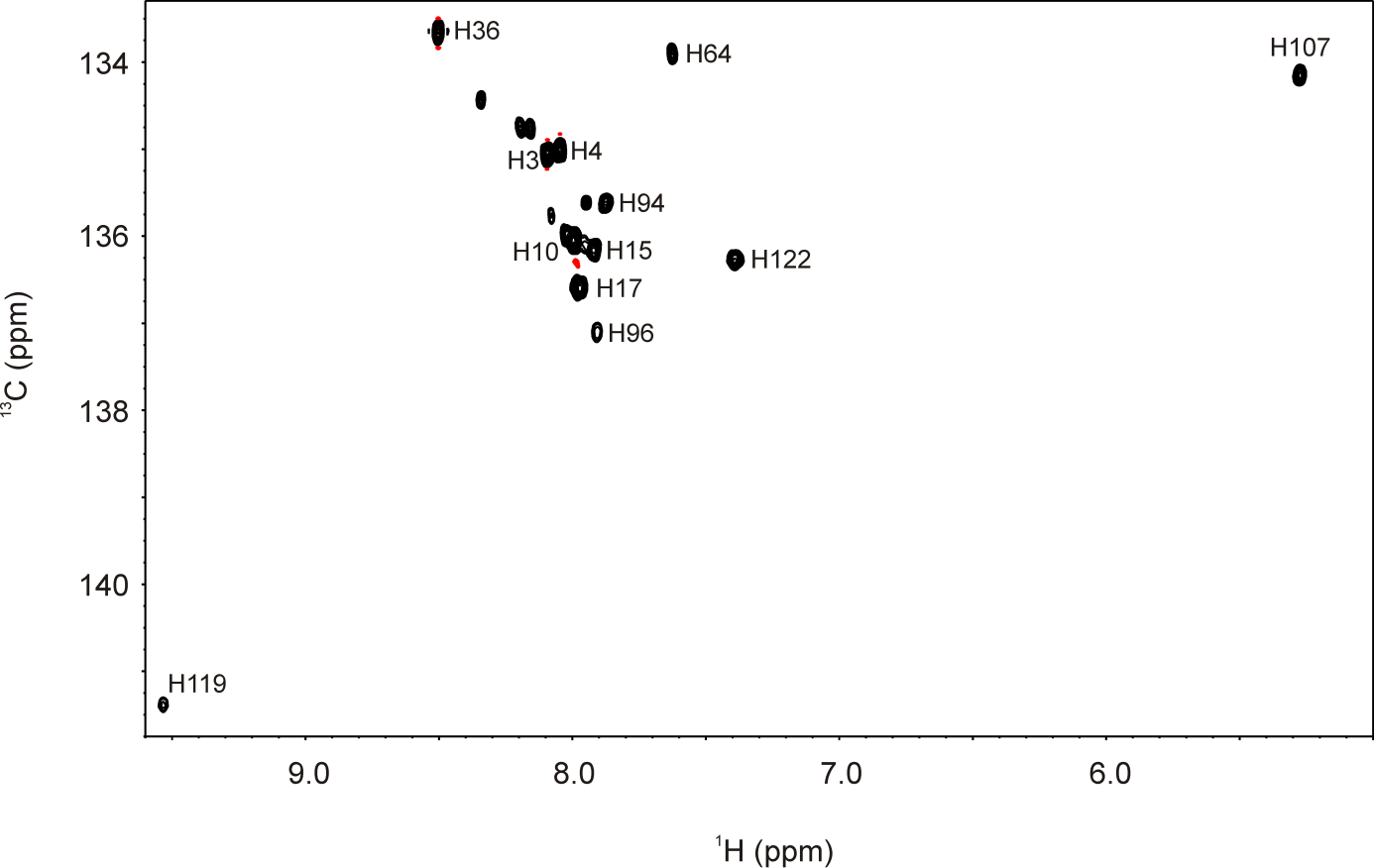
**

**Fig. S1** Hisε1 region of the aromatic ^1^H^13^C HSQC of WT HCA II. Signals are labeled according their assignment.

**
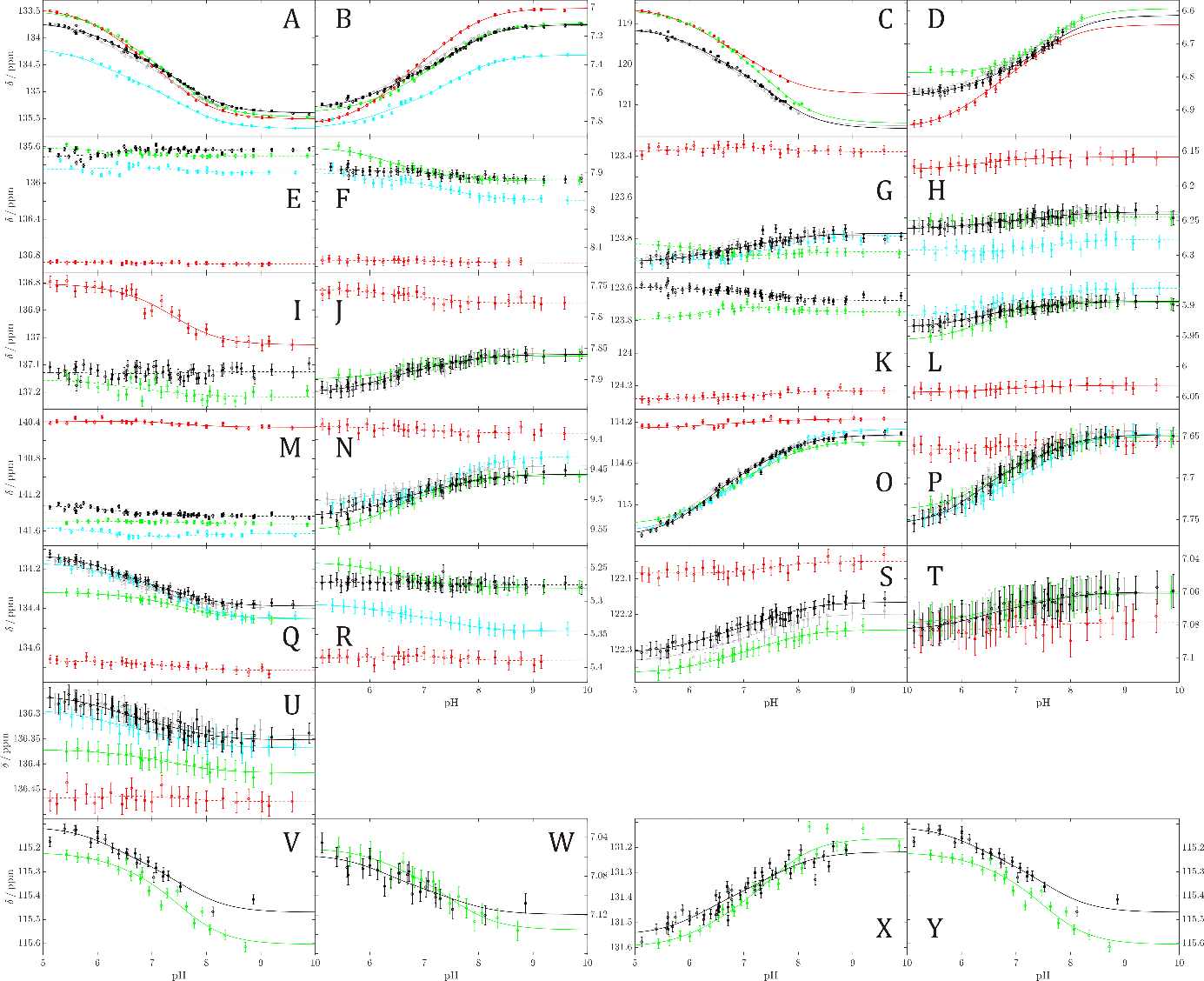
**

**Fig. S2 (part 1):** Experimental pH titration curves of H64 and its surrounding (W5, Y7, W16) and some inner positions (H94, H96, H119, H107, H122 and W209) for WT HCA II at 308K (black) and 314K (gray), with acetazolamide at 308K (red) and for the variants H4A (green) and Y7F (cyan) at 308K. First row from left to right: H64^13^Cε1 (A), H64^1^Hε1 (B), H64^13^Cδ2 (C) and H64^1^Hδ2 (D). Second row from left to right: H94^13^Cε1 (E), H94^1^Hε1 (F), H94^13^Cδ2 (G) and H94^1^Hδ2 (H). Third row from left to right: H96^13^Cε1 (I), H96^1^Hε1 (J), H96^13^Cδ2 (K) and H96^1^Hδ2 (L). Fourth row from left to right: H119^13^Cε1 (M), H119^1^Hε1 (N), H119^13^Cδ2 (O) and H119^1^Hδ2 (P). Fifth row from left to right: H107^13^Cε1 (Q), H107^1^Hε1 (R), H107^13^Cδ2 (S) and H107^1^Hδ2 (T). Sixth row: H122^13^Cε1 (U). Seventh row from left to right: Y7^13^Cε^*^ (V), Y7^1^Hε^*^ (W), Y7^13^Cδ^*^ (X) and Y7^1^Hδ^*^ (Y). Fits from the global fit are shown as solid lines, dashed lines were not included into the global fit. For the ^13^C data of H4A, a shift of around +0.1ppm is available through the non-use of linear prediction in the preprocessing which, however, has no effect on the calculated p*K*_a_ values.


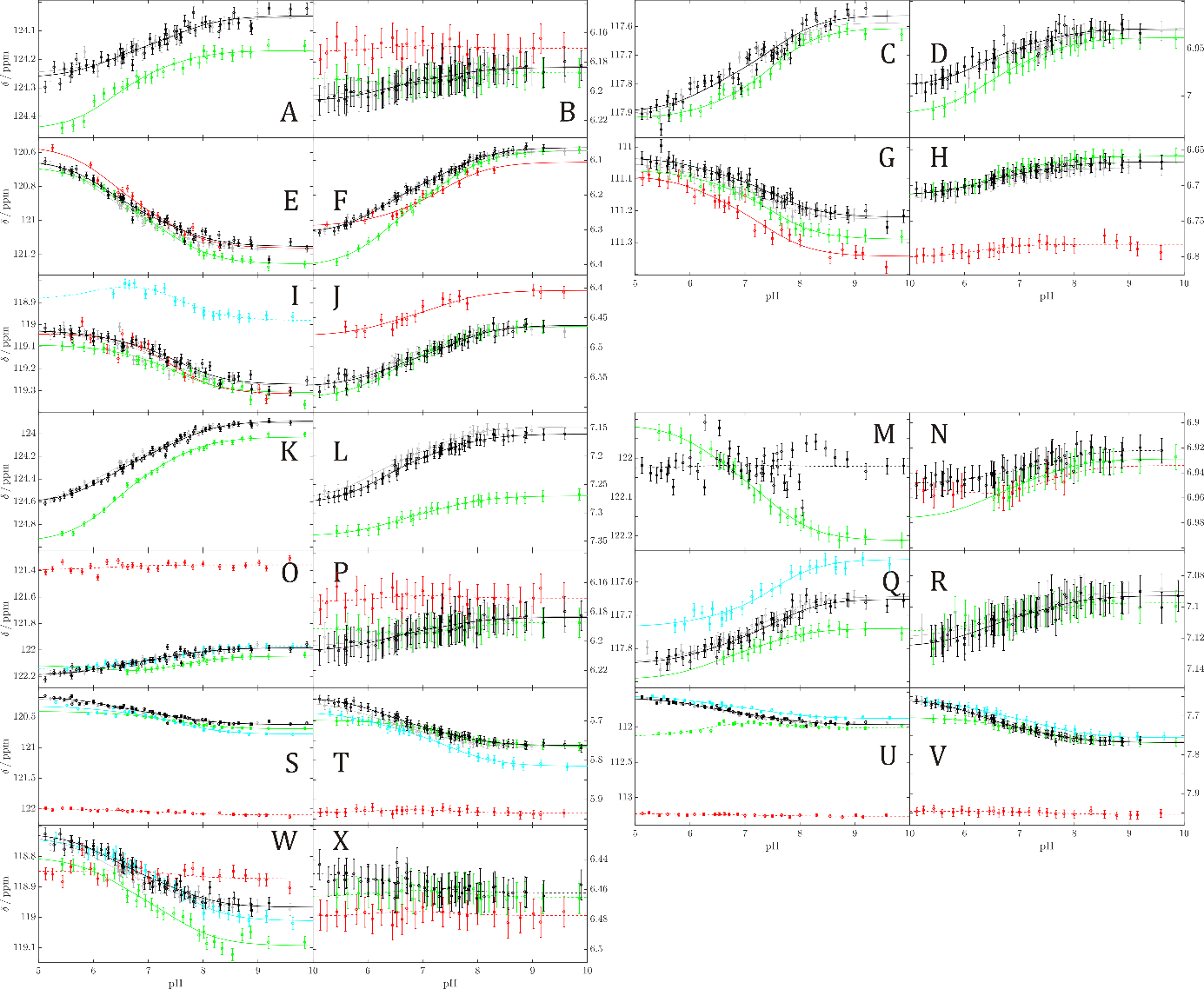


**Fig. S2 (part 2):** Experimental pH titration curves of H64 and its surrounding (W5, Y7, W16) and some inner positions (H94, H96, H119, H107, H122 and W209) for WT HCA II at 308K (black) and 314K (gray), with acetazolamide at 308K (red) and for the variants H4A (green) and Y7F (cyan) at 308K. First row from left to right: W5^13^Cδ1 (A), W5^1^Hδ1 (B), W5^13^Cε3 (C) and W5^1^Hε3 (D). Second row from left to right: W5^13^Cη2 (E), W5^1^Hη2 (F), W5^13^Cζ2 (G) and W5^1^Hζ2 (H). Third row from left to right: W5^13^Cζ3 (I) andW5^1^Hζ3 (J). Fourth row from left to right: W16^13^Cδ1 (K), W16^1^Hδ1 (L), W16^13^Cη2 (M) and W16^1^Hη2 (N). Fifth row from left to right: W209^13^Cδ1 (O), W209^1^Hδ1 (P), W209^13^Cε3 (Q) and W209^1^Hε3 (R). Sixth row from left to right: W209^13^Cη2 (S), W209^1^Hη2 (T), W209^13^Cζ2 (U) and W209^1^Hζ2 (V). Seventh row from left to right: W209^13^Cζ3 (W) andW209^1^Hζ3 (X). Fits from the global fit are shown as solid lines, dashed lines were not included into the global fit. For the ^13^C data of H4A, a shift of around +0.1ppm is available through the non-use of linear prediction in the preprocessing which, however, has no effect on the calculated p*K*_a_ values.

**
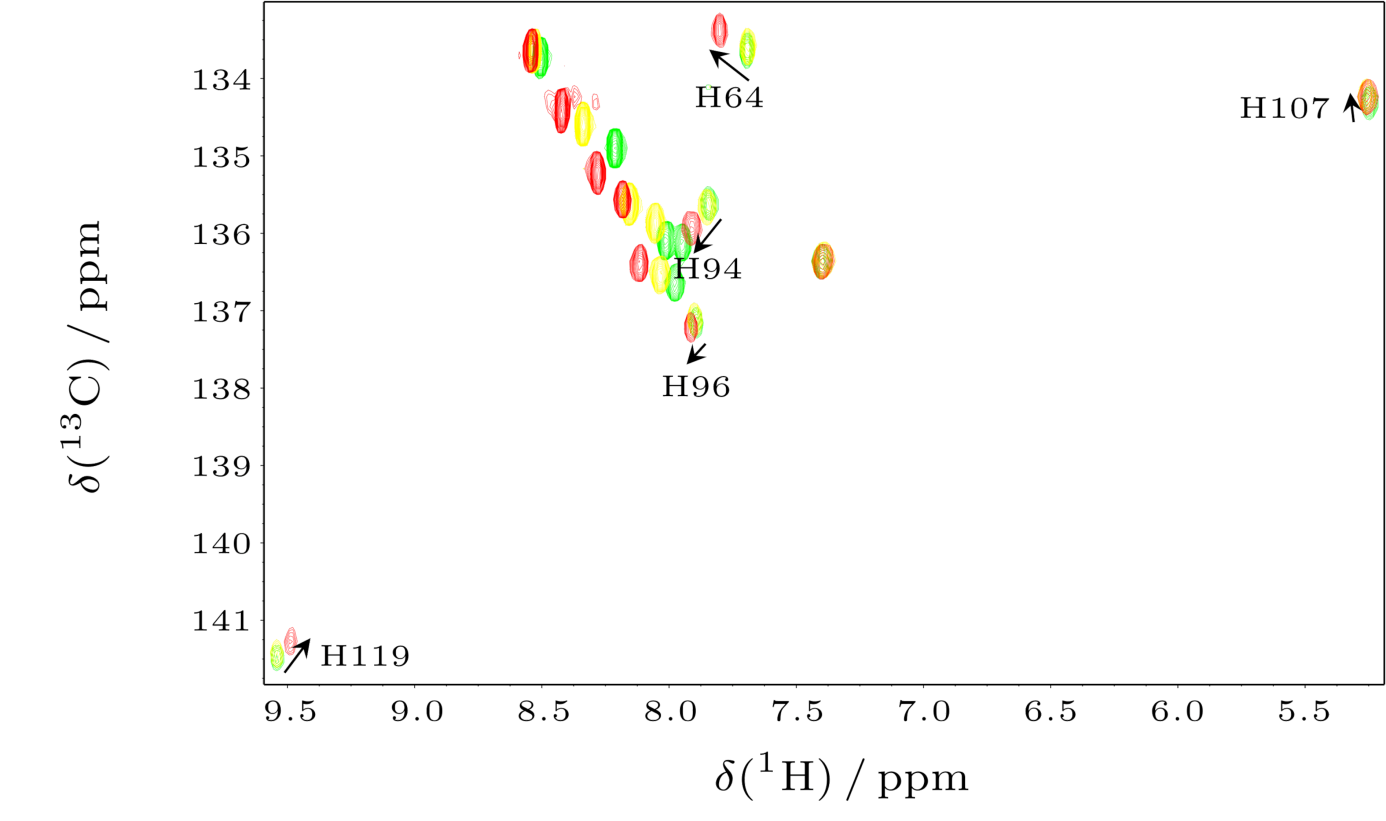
**

**Fig. S3** Hisε1 region of the aromatic ^1^H^13^C HSQC of the variantH4A HCA II at different pH (green 5.61; yellow 5.10; red 4.90). From a pH value approx. beneath 5.1, a jump in the chemical shift value occurs for the histidines near the center which cannot be explained by a normal titration behavior of a side chain.


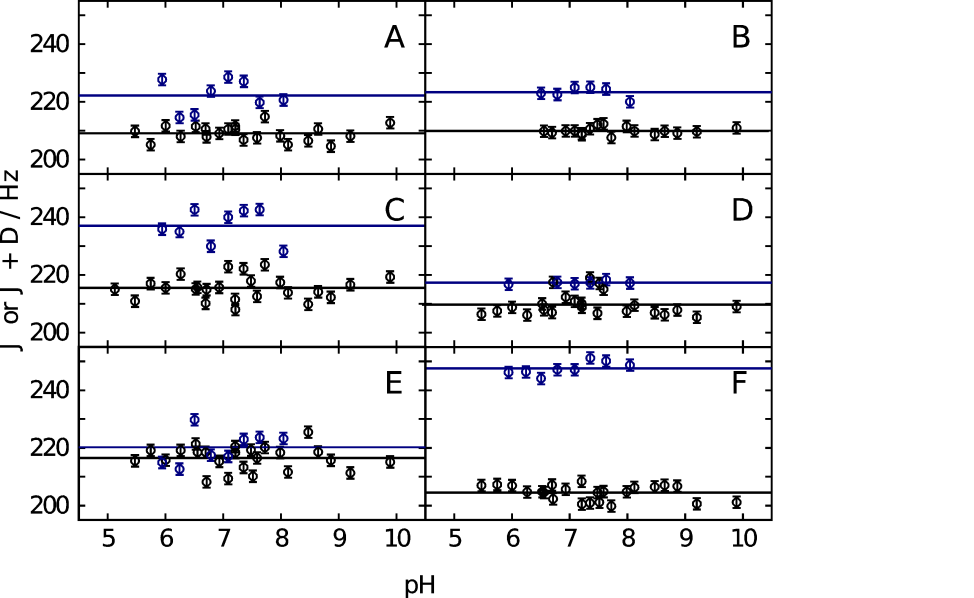


**Fig. S4** Measured ^1^*J*_1He1-13Ce1_-coupling values for WT HCA II wt at H94 (A), H17 (B), H96 (C), H107 (D), H119 (E) and H122 (F). The measured *J*-coupling in combination with the residual dipolar coupling *D* with alignment medium is shown in dark blue and solely J-coupling measured without alignment medium is shown in black. Mean values are displayed in the corresponding colour.

**Table S1**. Results from fitting the data to different models and *F*-statistics.

χ^2^values are from data fits to a monophasic transition (mono; 3 free parameters, no global fit and free p*K*_a_ value), to the hill model (hill; 4 free parameters, no global fit and free p*K*_a_ value), to a biphasic transition (l: low p*K*_a_ value of 6.25, w: water p*K*_a_ value of 6.8, h: high p*K*_a_ value of 7.60; 5 free parameters) or to a triphasic transition with all 3 p*K*_a_ values and 7 free parameters. For calculating *p*-values, model 1 vs. model 2 were compared and *F* was calculated by

$$F= \frac{(SS1-SS2)/(df1-df2)}{SS2/df2}$$

whereas *SS* is the sum of squares from the respective model and *df* the degree of freedom. The first model is the model with the higher degree of freedom. *p*-values are estimated from *F* and the degrees of freedom. For a large *p*-value, the first model is statistically better, for a low value, the second one.

| Residue | χ^2^(mono) | χ^2^(hill) | χ^2^(lw) | χ^2^(wh) | χ^2^(lh) | χ^2^(lwh) | N, datapoints | *p*-value | | | | | | | |
| --- | --- | --- | --- | --- | --- | --- | --- | --- | --- | --- | --- | --- | --- | --- | --- |
|  |  |  |  |  |  |  |  | mono vs. hill | mono vs. lw | mono vs. wh | mono vs. lh | mono vs. lwh | lw vs. lwh | wh vs. lwh | lh vs. lwh |
| H107Cδ2 | 14.7 | 9.2 | 16.5 | 11.6 | 8.6 | 8.4 | 52 | <0.00001 | 1 | 0.03 | <0.0001 | <0.0001 | <0.00001 | 0.0006 | 0.49 |
| H107Cε1 | 21.7 | 15.2 | 21.8 | 20.0 | 15.7 | 14.8 | 56 | <0.00002 | 1 | 0.38 | 0.003 | 0.0007 | <0.0001 | 0.0007 | 0.23 |
| H119Cδ2 | 232.6 | 63.2 | 206.9 | 230.5 | 68.7 | 68.3 | 58 | <0.00001 | 0.19 | 0.98 | <0.00001 | <0.00001 | <0.00001 | <0.00001 | 0.89 |
| H119Hδ2 | 10.9 | 6.8 | 9.6 | 12.4 | 6.1 | 5.8 | 58 | <0.00001 | 0.17 | 1 | <0.00001 | <0.00001 | <0.00001 | <0.00001 | 0.27 |
| H119Hε1 | 19.3 | 20.8 | 19.5 | 17.9 | 15.1 | 15.1 | 56 | 1 | 1 | 0.47 | 0.02 | 0.02 | 0.002 | 0.02 | >0.999 |
| H122Cε1 | 10.3 | 10.7 | 10.3 | 10.3 | 11.6 | 10.3 | 58 | 1 | 1 | >0.99 | 1 | >0.99 | 0.92 | 1 | 0.05 |
| H96Hδ2 | 6.5 | 5.7 | 6.1 | 7.8 | 5.1 | 5.0 | 58 | 0.006 | 0.47 | 1 | 0.01 | 0.007 | 0.005 | <0.00001 | 0.52 |
| H96Hε1 | 9.9 | 8.0 | 9.6 | 10.1 | 7.3 | 7.3 | 56 | <0.001 | 0.77 | 1 | 0.004 | 0.004 | 0.001 | 0.0003 | 0.94 |
| W209Cε3 | 31.1 | 29.8 | 42.6 | 29.2 | 29.0 | 28.7 | 51 | 0.16 | 1 | 0.59 | 0.52 | 0.47 | 0.0002 | 0.69 | 0.84 |
| W209Cη2 | 157.0 | 130.9 | 164.7 | 146.7 | 132.8 | 130.9 | 57 | 0.002 | 1 | 0.49 | 0.07 | 0.06 | 0.003 | 0.06 | 0.70 |
| W209Cζ2 | 58.1 | 48.0 | 57.8 | 56.3 | 52.4 | 48.7 | 53 | 0.002 | >0.99 | 0.83 | 0.30 | 0.08 | 0.02 | 0.04 | 0.19 |
| W209Cζ3 | 29.6 | 28.7 | 29.6 | 29.1 | 32.1 | 28.0 | 52 | 0.22 | >0.999 | 0.95 | 1 | 0.64 | 0.30 | 0.42 | 0.05 |
| W209Hη2 | 19.9 | 11.7 | 18.9 | 19.1 | 11.4 | 11.2 | 57 | <0.00001 | 0.61 | 0.71 | <0.0001 | <0.00001 | <0.00001 | <0.00001 | 0.63 |
| W209Hζ2 | 11.8 | 7.9 | 10.4 | 1.5 | 7.9 | 7.6 | 56 | <0.00001 | 0.18 | 1 | 0.0005 | 0.0002 | 0.0005 | <0.00001 | 0.38 |
